# Outbreak of *Yersinia enterocolitica* Serogroup O:9 Infection and Processed Pork, Norway

**DOI:** 10.3201/eid1305.061062

**Published:** 2007-05

**Authors:** Danica Grahek-Ogden, Barbara Schimmer, Kofitsyo S. Cudjoe, Karin Nygård, Georg Kapperud

**Affiliations:** * Norwegian Institute of Public Health, Oslo, Norway;; † National Veterinary Institute, Oslo, Norway; and; ‡ Norwegian School of Veterinary Science, Oslo, Norway

**Keywords:** *Yersinia enterocolitica*, serogroup 0:9, yersiniosis, outbreak, pork, dispatch

## Abstract

An outbreak involving 11 persons infected with *Yersinia enterocolitica* O:9 was investigated in Norway in February 2006. A case-control study and microbiologic investigation indicated a ready-to-eat pork product as the probable source. Appropriate control measures are needed to address consumer risk associated with this product.

Yersiniosis, which is reportable in Norway, is the third most commonly reported cause of acute enteritis after campylobacteriosis and salmonellosis. In the past 10 years, 80–150 cases of yersiniosis were registered annually; 1–10 of these cases were caused by serotype O:9. Most patients (70%–80%) acquire infection domestically (*1*). During January 2006, the National Reference Laboratory at the Norwegian Institute of Public Health (NIPH) received 6 human isolates of *Yersinia enterocolitica* O:9, which clearly exceeded the expected incidence. All of the infections were diagnosed within a 3-week period (*2*). A multidisciplinary outbreak investigation team was established to find the source and prevent further illness.

## The Study

A case-patient was defined as a resident of Norway with *Y. enterocolitica* O:9 isolated from stool or blood and with illness onset between December 15, 2005, to February 15, 2006. Cases were identified through the National Surveillance System at NIPH, which receives reports of laboratory-confirmed cases from laboratories and clinicians nationwide. Eleven cases of *Y. enterocolitica* O:9, biotype 2 infection were identified from December 21, 2005, through February 6, 2006 (Figure). Patients resided in 2 neighboring counties in southern Norway; all were adults except for a 10-year-old child. The median age was 44 years (range 10–88 years); 6 (54%) were female. Reported symptoms included severe abdominal pain, diarrhea, fever, arthralgia, and vomiting. Reactive arthritis developed in 1 patient. Symptoms lasted for a median of 14 days (9 days–6 weeks); 3 patients reported that symptoms had not resolved at the time of interview. Four patients were hospitalized, and 2 patients died; both were elderly with underlying medical conditions.

**Figure:**
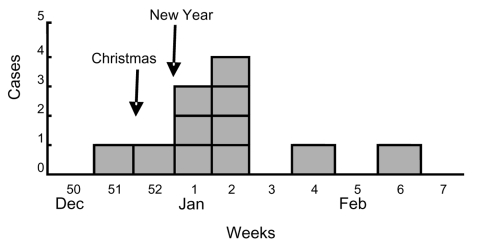
Distribution of patients with *Yersinia enterocolitica* 0:9 infection (n = 11) by week of onset, Norway, December 2005—February 2006.

The first 6 case-patients were interviewed by telephone, with a standard hypothesis-generating questionnaire requesting information on clinical symptoms and demographic data, in addition to food history, animal contacts, and environmental exposures in the 7 days before illness. On the basis of interview results, a case-control study was conducted that included 8 case-patients and 22 controls randomly selected from the national population register (ratio 1:3). Controls and case-patients were matched by age, sex, and municipality of residence. The median interval between illness onset and interview was 33 days (26–49 days), and case-patients and controls were interviewed on the same day. Controls were questioned about exposures in the week before illness of their matched case-patient. Matched odds ratios (mORs) from univariate analysis were calculated by using maximum-likelihood estimates, including 95% exact confidence intervals (CIs) by Fisher exact test (STATA 8.0, Stata Corporation, College Station, TX, USA). The same software was used for multivariate analysis by conditional logistic regression.

All isolates of *Yersinia* spp. from human patients were characterized phenotypically (*3*), biotyped (*4*), and serogrouped against absorbed rabbit antiserum produced at NIPH, representing O-antigen factors 1–34 (*5*). All suspected foods were examined for pathogenic *Y. enterocolitica* at the Norwegian Veterinary Institute by using 2 conventional cultivation methods (*6*,*7*). Isolates identified as *Y. enterocolitica* were forwarded to the National Reference Laboratory for verification and serotyping. Nested PCR targeting of chromosome-located virulence genes was also used (*8*).

In univariate analysis, eating processed pork product (“julesylte,” Christmas brawn) was associated with *Y. enterocolitica* O:9 infection (mOR 6.7, 95% CI 0.7–64.1). Patients were also more likely than controls to have eaten pork chops (mOR 5.9, 95% CI 0.6–61.3) (Table 1). Consumption of brawn and consumption of pork chops were both independently related to increased risk in conditional logistic regression analysis. On February 10, the public health and food safety authorities issued a public warning advising consumers against eating brawn.

**Table 1:** Selected exposures of patients with *Yersinia enterocolitica* 0:9 infection and matched controls, univariate analysis, Norway, February 2006*

Food exposure	Case-patients, N = 8 Exposed/total (%)	Controls, N = 22 Exposed/total (%)	Matched OR (95% CI)
Brawn	5/8 (63)	5/22 (23)	6.7 (0.7–64.1)
Pork chops	4/7 (57)	5/20 (25)	5.9 (0.6–61.3)
Wiener sausage	5/7 (71)	13/21 (62)	1.6 (0.2–10.3)
Smoked sausage	3/8 (38)	7/21 (33)	1.2 (0.2–6.0)
Salami	3/8 (38)	6/22 (27)	1.2 (0.1–11.3)
Pork burgers	3/6 (50)	10/20 (50)	1.2 (0.2–8.0)
Roulade	2/8 (25)	4/21 (19)	1.1 (0.2–6.3)
Ribs	4/8 (50)	11/21 (52)	0.9 (0.2–5.0)
Saveloy	2/7 (29)	6/21 (29)	0.9 (0.1–6.0)

*OR, odds ratio; CI, confidence intervals.

The case-control study did not identify specific producers. Consequently, products were sampled on the basis of tracing of product deliveries to implicated shops. Sixty-two samples were examined from major producers and small local butchers; the samples comprised 54 brawn (3 obtained from opened packages from patients’ homes, 9 from opened and sliced batches at retailers), 1 cooked ham, and 7 other pork products from patients’ homes (Table 2). None yielded pathogenic *Yersinia* organisms by culture methods. However, nonpathogenic *Y. enterocolitica* or *Y. intermedia* strains were isolated from 6 (10%) samples. Positive PCR results were obtained for 20 (32%), including 16 brawn samples from patients’ homes and retailers. Eleven of the 20 positive brawn from unopened packages came from 6 different producers. To ascertain whether positive PCR results were from viable or dead target bacteria, colonies from enrichment cultures were retested by nested PCR. Nineteen colonies, including those originating from brawn from patients’ homes, were positive. DNA sequence analysis of the PCR product from 1 brawn from a patient’s home showed 321 of 322 base pairs matched when compared with the PCR product obtained from 1 random patient isolate.

**Table 2:** Results from PCR analyses of pork products sampled during investigation of *Yersinia enterocolitica* O:9 outbreak, Norway, 2006

Sample types	No. samples	Positive		Negative	
		16	Colony swabs	Broth culture	Colony swab
Brawn	54	1	16	38	38
Cooked ham	1	3	1	0	0
Other pork products	7	20	2	4	5
Total	62				

No other public health measures were introduced because brawn is sold and consumed only at Christmas. Other, long-term measures—including establishing levels of contamination of raw materials and ready-to-eat pork products, assessment of production processes, and establishing reliable microbiologic methods—were suggested.

## Conclusions

To our knowledge, this investigation confirmed for the first time both an outbreak of *Y. enterocolitica* O:9 in Norway and a ready-to-eat pork product associated with an outbreak. Both epidemiologic and microbiologic findings suggested brawn as the probable source.

Several studies have found pork consumption to be associated with yersiniosis (*9*–*11*). Risk factors for sporadic *Y. enterocolitica* infections were also assessed in a prospective case-control study in Norway in 1988–1990 (*12*). Results from that study showed that infected persons ate significantly more pork products and sausages than did matched controls. Case-patients were also more likely than controls to prefer raw or rare meat and to drink untreated water. All factors were independently associated with disease. Because of the importance of pork products in yersiniosis epidemiology, attempts were made to identify hazards in swine slaughter so that appropriate preventive measures could be defined (*13*). Measures implemented at farm herd level, in abattoirs, and during meat processing were suggested to minimize contamination of retail pork products (*14*). Introduction of these measures has resulted in considerable reduction in contamination and a decrease of human yersiniosis in Norway (*15*).

Although the case-control study suggested brawn as the outbreak source, it also showed an association with eating pork chops. The power of the case-control study was limited by the small number of cases. However, in-depth interviews with 10 of 11 patients—as well as with patients not included in the case-control study—showed that all had eaten brawn.

Brawn is traditional at Christmas. It is prepared by layering pork meat (precooked head muscles), veal, lard, and spices in a mold. The brawn mold is cooked to an assumed core temperature of 74°C before the temperature is reduced and the meat is maintained at a temperature of 70°C for at least 30 min. After removal from the mold, the brawn is vacuum-packed for sale—usually whole, although some producers package sliced products. At some delicatessen counters, packages might be opened and products sliced. Some people also prepare brawn at home.

Correct cooking should eliminate *Y. enterocolitica,* but microbiologic analyses have shown bacteria can survive in the product core. Lard, with its high fat content, can enhance survival of the bacteria. Furthermore, cross-contamination can occur if good hygiene procedures are not strictly observed when the brawn is removed from the mold before packaging. At that point, contamination is superficial, but slicing would spread microorganisms throughout the product. The ability of *Y. enterocolitica* to grow at refrigeration temperatures gives it an advantage over other microorganisms and may further compound the problem.

Despite existing control measures, this outbreak demonstrated that ready-to-eat processed meats may represent an important risk for consumers to acquire *Yersinia* infection. Producers need to be reminded of their responsibility to ensure safe products, since consumers cannot always take precautions. Research to clarify the role of pork meat in *Yersinia* epidemiology and improved laboratory methods for detecting pathogenic *Y. enterocolitica* in food are needed.
